# TRAF3 Regulates Homeostasis of CD8^+^ Central Memory T Cells

**DOI:** 10.1371/journal.pone.0102120

**Published:** 2014-07-10

**Authors:** Zuoan Yi, Laura L. Stunz, Wai Wai Lin, Gail A. Bishop

**Affiliations:** 1 Department of Microbiology, University of Iowa, Iowa City, Iowa, United States of America; 2 Graduate Immunology Program, University of Iowa, Iowa City, Iowa, United States of America; 3 Department of Internal Medicine, University of Iowa, Iowa City, Iowa, United States of America; 4 VA Medical Center, Iowa City, Iowa, United States of America; New York University, United States of America

## Abstract

Our laboratory reported previously that TNF receptor associated factor 3 (TRAF3) is a positive regulator of TCR signaling and T cell function. In the current study, we present new findings that reveal differential roles for TRAF3 in the regulation of CD4^+^ and CD8^+^ T cells. In response to TCR stimulation *in vitro*, TRAF3 has greater impact in CD4^+^ T cells than in CD8^+^ T cells. However, T cell-specific TRAF3 deficient mice (CD4^Cre^ TRAF3^fl^°^x/fl^°^x^; T-TRAF3^−/−^) have a greater number of CD4^+^CD44^hi^ effector/memory T cells than littermate control (LMC) mice, possibly due to an inefficient suppressive effect of TRAF3 deficient Foxp3^+^ regulatory T cells. In contrast, CD8^+^CD44^hi^CD62L^hi^ central memory (Tcm) cells are markedly reduced in T-TRAF3^−/−^ mice in comparison to LMC mice, although CD8^+^CD44^hi^CD62L^l^°^w^ effector memory T (Tem) cells and naïve T cells (CD8^+^CD44^l^°^w^CD62L^hi^) do not show significant differences in number. Importantly, TRAF3-deficient Tcm cells exhibit defective homeostasis due to impaired IL-15 signaling. These results indicate that the involvement of TRAF3 in IL-15 mediated signaling to T cells plays a previously unappreciated and critical role in CD8^+^ Tcm cell regulation and maintenance.

## Introduction

T cell-mediated immunity includes activation of naïve T cells through TCR: MHC-peptide engagement, various functions performed by activated effector T cells, and persistence of antigen-specific memory T cells. The development of long-lasting memory T cell responses is the basis for immunization and vaccination, as such responses also support the function and activation of memory B lymphocytes. Memory T cells differentiate from naïve T cells after antigenic stimulation and can be divided into two main subsets: effector memory (Tem) and central memory (Tcm) cells. Tem cells (CD44^hi^CD62L^l^°^w^CCR7^l^°^w^) circulating in non-lymphoid organs can respond rapidly after encountering pathogens or infected cells. In contrast, Tcm cells (CD44^hi^CD62L^hi^CCR7^hi^) residing in secondary lymphoid organs have greater proliferative potential than Tem cells [1]–[6]. Although antigen, co-stimulation and cytokines contribute to the formation of diverse types of effector and memory T cells, maintenance of memory T cells mainly requires signals through common cytokine γ–chain receptors (γ_c_) [7]–[9]. The numbers of memory T cells do not show considerable change with time, indicating a relatively stable homeostatic proliferation rate of this population [10]. Understanding of the molecular mechanisms that regulate the development and maintenance of the memory population will open new avenues for designing novel strategies for immunization and vaccination.

TNF receptor associated factor 3 (TRAF3) is an adaptor molecule that participates in signaling by many members of the TNF receptor superfamily, as well as innate immune receptors and the IL-17 receptor [11]–[13]. Mice with a germline deletion of the *traf3* gene die within 2 weeks of birth [14]. Recent studies indicate that the role of TRAF3 is highly receptor and cell type-dependent. TRAF3 is required for production of type I interferon and IL-10 induced by Toll-like receptors (TLR) in macrophages and dendritic cells [15], [16]. However, in B cells TRAF3 plays a negative role in CD40 and TLR mediated signaling, and downstream antibody production [17], [18]. Deficiency of TRAF3 results in prolonged survival of B cells but not T cells, although both cell types display enhanced non-canonical NF-κB2 activation in the absence of TRAF3 [19]–[21]. TRAF3 also negatively regulates IL-17R signaling in myeloid cells [22]. Our recent studies reveal that T cell-specific deficiency in TRAF3 causes defective development and function of invariant Natural Killer T (iNKT) cells [23]. Additionally, a recent study indicates that Foxp3^+^ regulatory T (Treg) cell-specific TRAF3 expression is required for follicular Treg cell induction [24]. Using our T cell-specific TRAF3 deficient mouse model (T-TRAF3^−/−^), we also found that T cell effector functions are defective and TCR signaling impaired in peripheral T cells. CD3+CD28-stimulated cytokine production of CD4^+^ T cells *in vitro* is also severely impaired. However, in contrast, cytokine production by CD8^+^ T cells is only moderately affected by the absence of TRAF3. In addition, increased levels of T cell death occur in TRAF3-deficient T cells following TCR stimulation. Enhanced apoptosis as well as decreased TCR complex signaling could contribute to the substantially reduced cytokine production of TRAF3^−/−^ T cells [21]. Thus, a remaining knowledge gap is to what extent defects in TCR signaling versus additional TRAF3-dependent events contribute to altered CD4^+^ and CD8^+^ T cell functions in T-TRAF3^−/−^ mice. Studies summarized above point to the multifaceted nature of TRAF3 in regulating immune cell functions [25].

Findings presented here reveal differences in the regulatory roles of TRAF3 in CD4^+^ and CD8^+^ T cells. In response to *in vitro* TCR stimulation, only TRAF3 deficient CD4^+^ T cells, but not CD8^+^ T cells show defective early activation. Interestingly, T-TRAF3^−/−^ mice exhibit more CD4^+^CD44^hi^ effector/memory T cells than LMC mice. In contrast, there are remarkably fewer CD8^+^ Tcm cells in T-TRAF3^−/−^ mice, despite relatively normal numbers of Tem cells and naïve T cells. Results in this study reveal a TRAF3-dependence of IL-15 signaling to Tcm cells that may underlie this deficiency.

## Materials and Methods

### Mice

TRAF3^fl^°^x/fl^°^x^ mice were described previously [19] and backcrossed with C57BL/6 mice for 10 generations. TRAF3^fl^°^x/fl^°^x^ mice were bred with CD4^Cre^ mice as before [21]. Mice of 6–12 wk of age were used for all experiments. Age-matched T-TRAF3^−/−^ and LMC mice were euthanized through CO_2_ inhalation followed by cervical dislocation for each experiment. All mice were maintained in facilities under specific pathogen-free conditions at The University of Iowa and were used in accordance with National Institutes of Health guidelines under an animal protocol approved by the Animal Care and Use Committee of the University of Iowa.

### Flow cytometry

Single-cell suspensions were prepared from spleens or lymph nodes, and erythrocytes were lysed. For flow cytometry staining, cells were blocked with anti–mouse CD16/CD32 mAb and stained with fluorescently labeled antibodies against CD4 (L3T4), Foxp3 (FJK-16s), CD8α (53–6.7), CD44 (IM7), CD62L (MEL-14), CD69 (H1.2F3), CD25 (eBio7D4), CD122 (TM-b1) and CCR7 (4B12). All antibodies were purchased from eBioscience (San Diego, CA). Flow cytometric analysis and cell sorting were performed using a FACS LSRII or Aria (BD) at The University of Iowa Flow Cytometry Facility. Results were analyzed with FlowJo software (Tree Star).

### Cytokine detection

Splenocytes were stimulated with PMA (10 ng/ml) and ionomycin (0.5 µg/ml) in the presence of Brefeldin A (10 µg/ml) (Sigma Aldrich, St. Louis, MO) for 6 hr. Surface staining for CD4, CD8, CD44 and CD62L was performed followed by intracellular staining for IL-2, TNF-α, IL-17, IL-10 and IFN-γ (eBioscience) using Cytofix/Cytoperm reagents (BD Bioscience, San Jose, CA). Cells were analyzed by flow cytometry.

### IL-7 and IL-15 receptor signaling

Splenocytes were incubated with recombinant mouse IL-7 (10 ng/ml) or IL-15 (20 ng/ml) (Peprotech, Rocky Hill, NJ) for 20 min. Cells were fixed immediately with 2% paraformaldehyde for 10 min at room temperature and permeabilized with cold methanol for 20 min. Surface staining was performed with anti-CD4, CD8, CD44 and CD62L Abs after washing. Phosphorylation of STAT5, ERK and S6K was detected with anti-p-STAT5, p-ERK and p-S6K Ab (Cell Signaling Technology, Danvers, MA) followed by Alexa647-conjugated anti-rabbit secondary Ab. Cells were analyzed by flow cytometry.

### T cell proliferation assays

Purified CD8^+^ T cells (CD8^+^ T cell isolation kit, Miltenyi Biotec, Auburn, CA) were labeled with 5 µM CFSE (Sigma) and seeded at 2×10^5^ cells/well. For cytokine stimulation, CD8^+^ T cells were stimulated with IL-15 (60 ng/ml) for 72 hr. Cells were stained with fluorescently-labeled Abs specific for CD8, CD44 and CD62L. CFSE dilution was evaluated by flow cytometry.

### Detection of apoptosis

Mouse splenocytes, freshly isolated or cultured for 24 hr without stimulation were stained for expression of CD8, CD44, CD62L and Annexin V. Relative levels of Annexin V positive cells in different populations were detected by flow cytometry.

### Statistical analysis

Data represent mean ± SEM of replicate samples, as indicated in Figure legends. Statistical comparisons of differences between sample means were performed using Student’s t test.

## Results

### Impact of TRAF3 upon responses of CD4^+^ and CD8^+^ T cells to *in vitro* stimulation via the TCR

**Figure 1:**
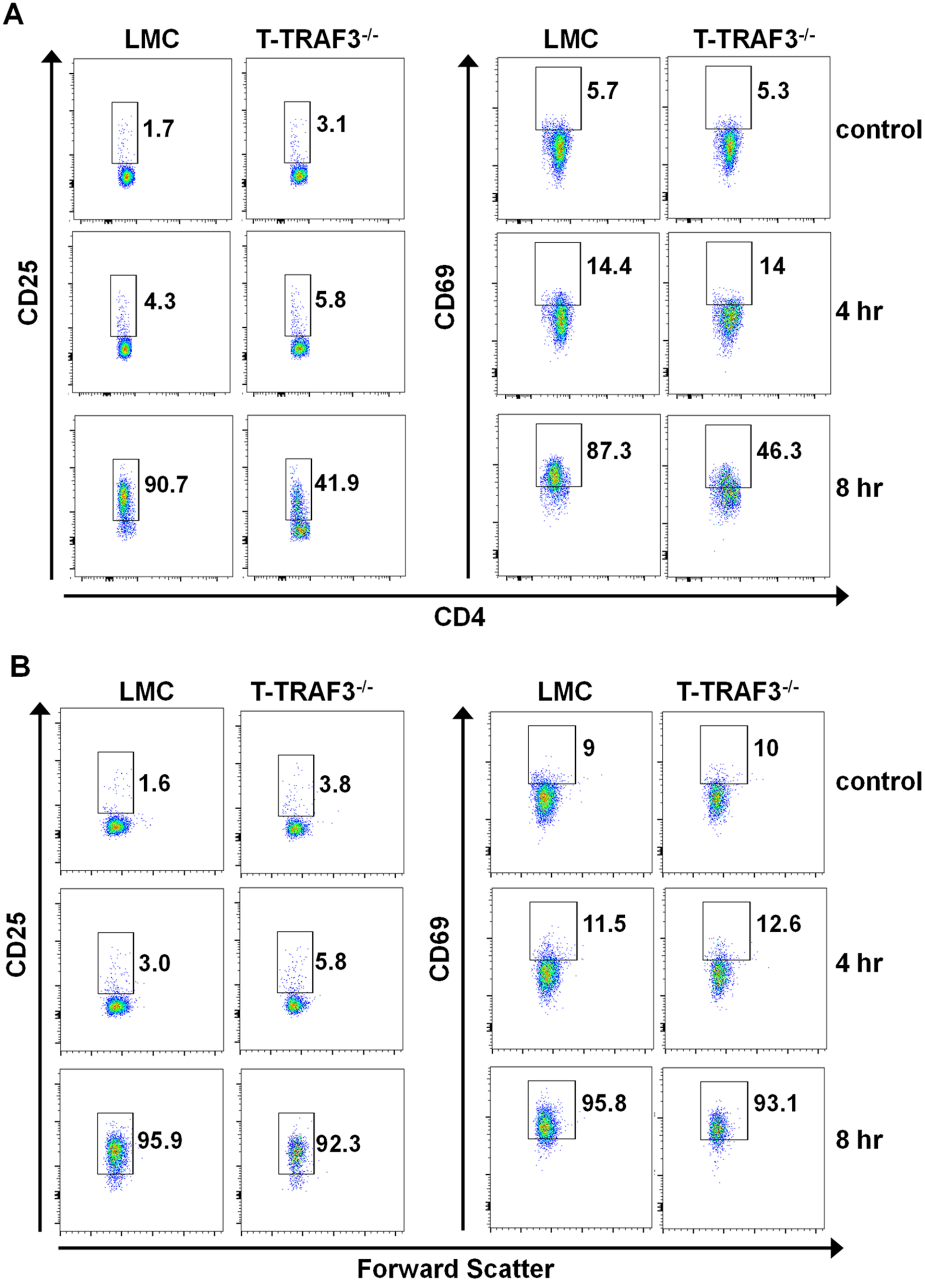
Effect of TRAF3 status upon TCR-mediated *in vitro* activation of CD4^+^ and CD8^+^ T cells. Sorted naïve CD4^+^ (CD4^+^CD25^−^CD44^l^°^w^CD62L^hi^) and CD8^+^ T cells (CD8^+^CD44^l^°^w^CD62L^hi^) were stimulated with anti-CD3 (1 ug/ml) and anti-CD28 (5 ug/ml) Abs for the indicated times. CD25 and CD69 expression was assessed by flow cytometric analysis. CD4^+^ T cells and CD8^+^ T cells are shown in (**A**) and (**B**), respectively. Data represent one of three individual experiments.

We previously reported defective TCR signaling and considerably impaired T cell responses to immunization and bacterial infection in mice with loss of TRAF3 specifically in T cells. In the case of *in vitro* TCR-stimulated cytokine production, however, the defect seen in CD4^+^ T cells was markedly more severe than that observed in CD8^+^ T cells of the T-TRAF3^−/−^ mouse [21]. Given the diverse roles played by TRAF3 in different cell subsets and in signaling by distinct receptors, we sought to explore whether TRAF3 plays differential roles in regulating CD4^+^ versus CD8^+^ T cells. To address this question, purified naive CD4^+^ and CD8^+^ T cells were stimulated with anti-CD3 and CD28 Abs and early activation markers were assessed. Results (Figure 1A) show that ∼90% of LMC CD4^+^ T cells expressed CD25 and CD69 after 8 hr stimulation. However, only ∼40% of TRAF3^−/−^ CD4^+^ T cells were CD25 and CD69 positive, consistent with the weakened TCR signaling in CD4^+^ T cells [21]. In striking contrast to CD4^+^ T cells, in CD8^+^ T cells these activation markers were equally upregulated in both LMC and TRAF3^−/−^ T cells (Figure 1B). These results indicate that TRAF3 is more important to early TCR+CD28 signaling in CD4^+^ T cells than in CD8^+^ T cells.

### Effector/memory T cells in T-TRAF3^−/−^ mice

**Figure 2:**
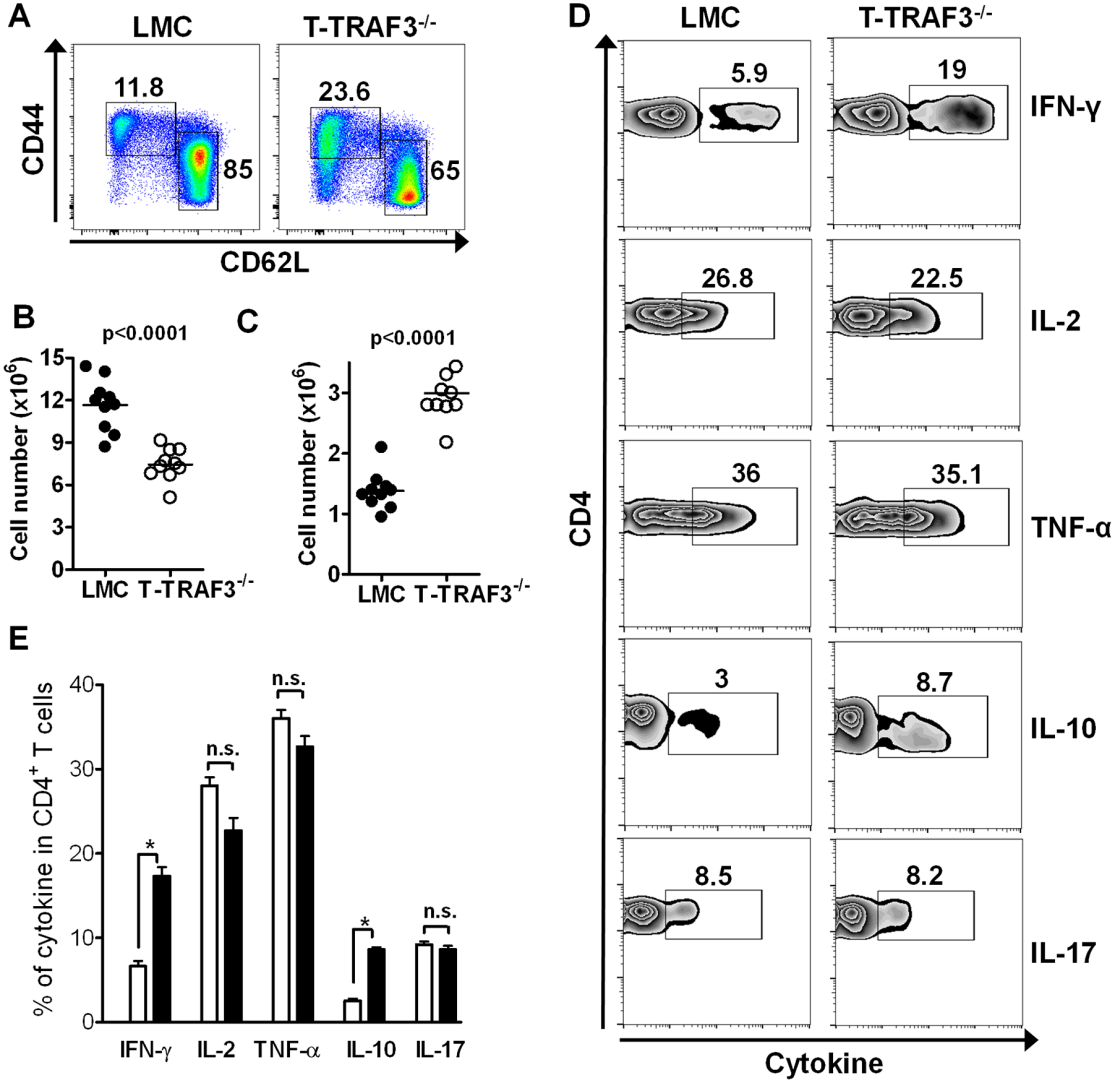
Increased effector/memory CD4^+^ T cells in T-TRAF3^−/−^ mice. Splenocytes were stained for CD4, Foxp3, CD44 and CD62L. CD4^+^Foxp3^−^ T cells were gated for CD44 and CD62L analysis. (**A**) percentages, (**B**) numbers of naïve and (**C**) numbers of CD44^hi^CD62L^l^°^w^ effector/memory T cells are shown. Data represent 10 mice in each group. (**D**) and (**E**) Splenocytes were stimulated with PMA and ionomycin for 6 hr. Intracellular staining for cytokine production in CD4^+^ T cell subsets was performed. (**E**) Summarized data from three individual experiments. *p<0.01.

To further explore how TRAF3 deficiency affects T cell activation status *in vivo*, we measured the effector/memory population in CD4^+^ and CD8^+^ T cells. Flow cytometric analysis showed that splenic effector/memory T cells (CD44^hi^CD62L^l^°^w^) in CD4^+^Foxp3^−^ T cells were approximately doubled in the absence of TRAF3, and the naïve T cell population (CD44^l^°^w^CD62L^hi^) was correspondingly reduced (Figure 2A–C). These results were reproduced in lymph node T cells (data not shown). To explore the intrinsic ability of TRAF3-deficient CD4^+^ T cells to produce cytokines, we bypassed TCR signaling by stimulating T cells with PMA + ionomycin. Under these conditions, TRAF3-deficient CD4^+^ T cells produced significantly more IFN-γ and IL-10 compared to LMC CD4^+^ T cells, but similar amounts of TNF-α, IL-2 and IL-17 (Figure 2D and E). Thus, the previously reported defect in TCR-mediated cytokine production can be attributed to altered TCR signaling in the absence of TRAF3. Interestingly, mice with TRAF3 depleted only from CD4^+^Foxp3^+^ Treg cells also showed increased CD4^+^ effector/memory cells, indicating that TRAF3 expressed in Treg cells is required for suppressing CD4^+^ effector/memory cells [24]. Therefore, the increase in effector/memory CD4^+^ T cells in T-TRAF3^−/−^ mice is due to cell extrinsic effects.

**Figure 3:**
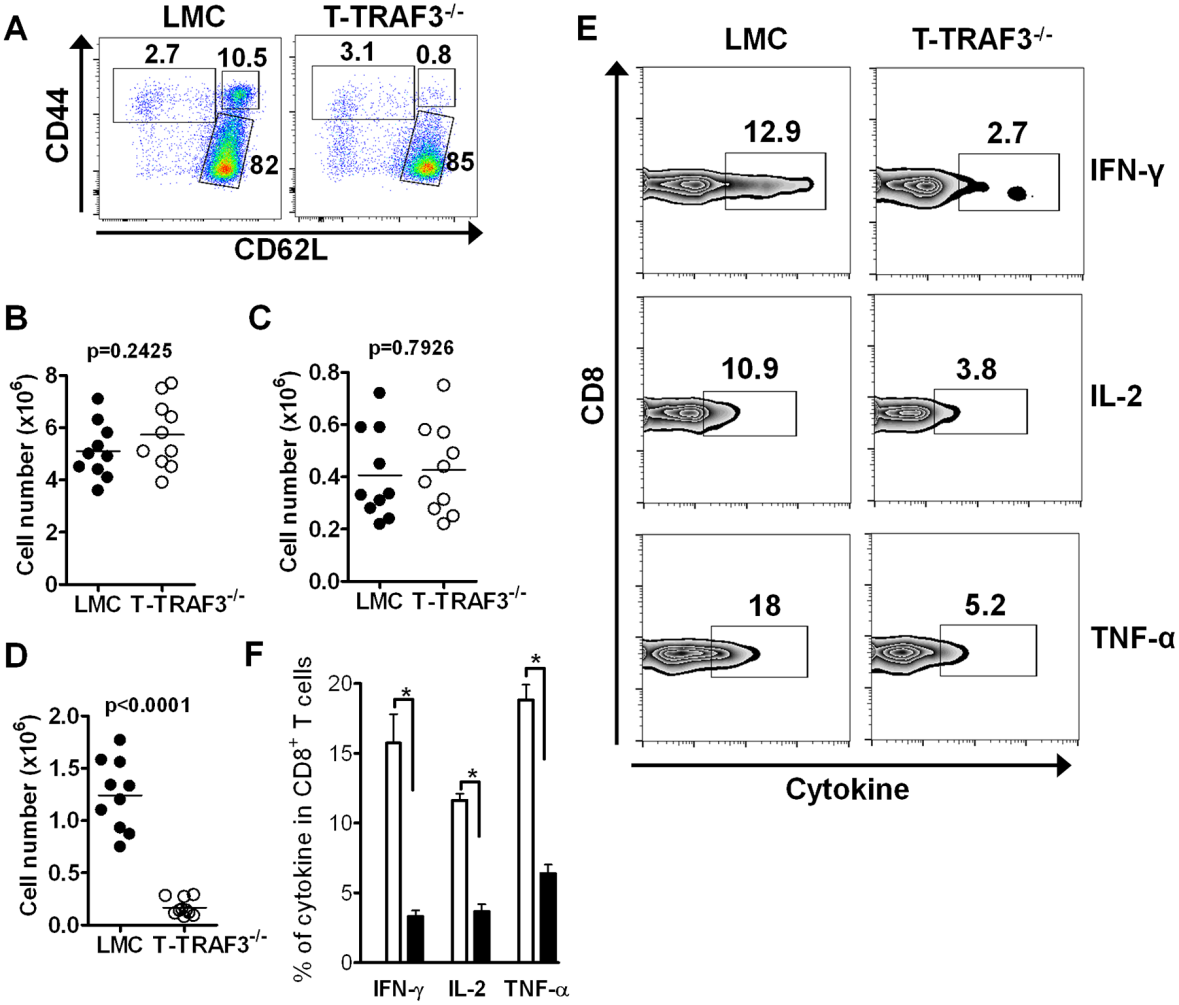
Reduced CD8^+^ central memory T cells in T-TRAF3^−/−^ mice. Splenocytes were stained for CD8, CD44 and CD62L. (**A**) Percentages of CD8^+^ naïve, Tem and Tcm cells in LMC and T-TRAF3^−/−^ mice. Numbers of naïve (**B**), Tem (**C**) and Tcm (**D**) cells are shown. (**E**) and (**F**) Splenocytes were stimulated with PMA and ionomycin for 6 hr. Intracellular staining for cytokine production in CD8^+^ T cell subsets was performed. (**F**) Summarized data from three individual experiments. *p<0.01.

Different CD8^+^ T cells subsets can be distinguished by the expression of CD44, CD62L, CCR7 and/or CD122. To explore how TRAF3 deficiency affects CD8^+^ T cells *in vivo*, we examined distinct subsets of CD8^+^ T cells. The percentage and number of Tem cells (CD44^hi^CD62L^l^°^w^) and naïve T cells (CD44^l^°^w^CD62L^hi^) were comparable between T-TRAF3^−/−^ and LMC mice (Figure 3A–C). However, Tcm cells (CD44^hi^CD62L^hi^) were reduced 5–10 fold in the absence of TRAF3 (Figure 3A and D). This was also true when cells were stained for CD122 and CCR7 (data not shown). When stimulated with PMA + ionomycin, TRAF3-deficient CD8^+^ T cells produced much less IFN-γ, IL-2 and TNF-α (Figure 3E and F), consistent with the smaller population of memory CD8^+^ T cells. Taken together, these results indicate that TRAF3 deficiency in CD8^+^ T cells largely affects the generation and/or maintenance of Tcm cells.

### Impaired IL-15 signaling in TRAF3-deficient CD8^+^ Tcm cells

**Figure 4:**
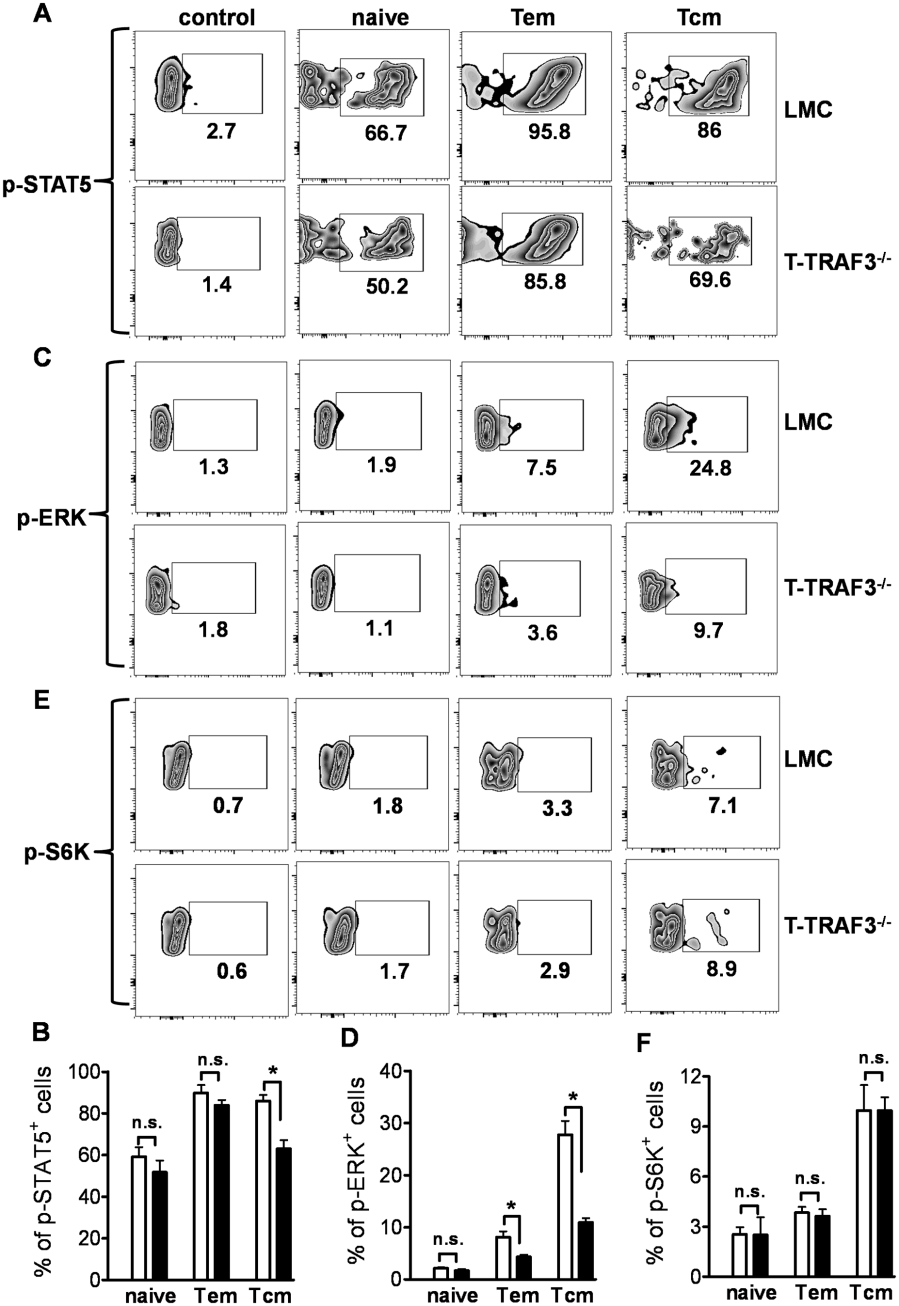
Impaired IL-15 signaling in TRAF3-deficient CD8^+^ Tcm cells. Splenocytes were stimulated with 20/ml IL-15 for 20 min. Cells were fixed, permeabilized and surface-stained for CD8, CD44 and CD62L. Phosphorylation of STAT5 (**A**), ERK (**C**) and S6K (**E**) was detected through intracellular staining and FACS analysis. Percentage of cells with phospho-STAT5 (**B**), -ERK (**D**) and -S6K (**F**) is summarized from three experiments. *p<0.01.

The homeostasis of CD8^+^ Tcm cells relies primarily upon IL-7 and IL-15, but not TCR signaling [4]–[6]. The reduction in the CD8^+^ Tcm population prompted us to hypothesize that IL-7 and/or IL-15 signaling was impacted by the absence of TRAF3. To test this hypothesis, CD8^+^ T cells were stimulated with IL-7 or IL-15 and early signaling events were examined. Upon IL-15 stimulation, the phosphorylation of the transcriptional regulator STAT5 and the MAP kinase ERK in Tcm cells were greatly reduced in the absence of TRAF3, but the phosphorylation of the kinase S6K was not affected (Figure 4A–F). In CD8^+^ Tem cells, only phosphorylation of ERK was notably reduced in the absence of TRAF3. No differences in IL-15 signaling were observed in naïve T cells lacking TRAF3 (Figure 4A–F). No alterations in IL-7 responses were seen in TRAF3^−/−^ CD8^+^ T cells (data not shown). These results indicate that TRAF3 is required for IL-15 mediated activation of STAT5 and ERK signaling pathways in Tcm cells.

### Defective homeostasis of CD8^+^ Tcm cells in the absence of TRAF3

**Figure 5:**
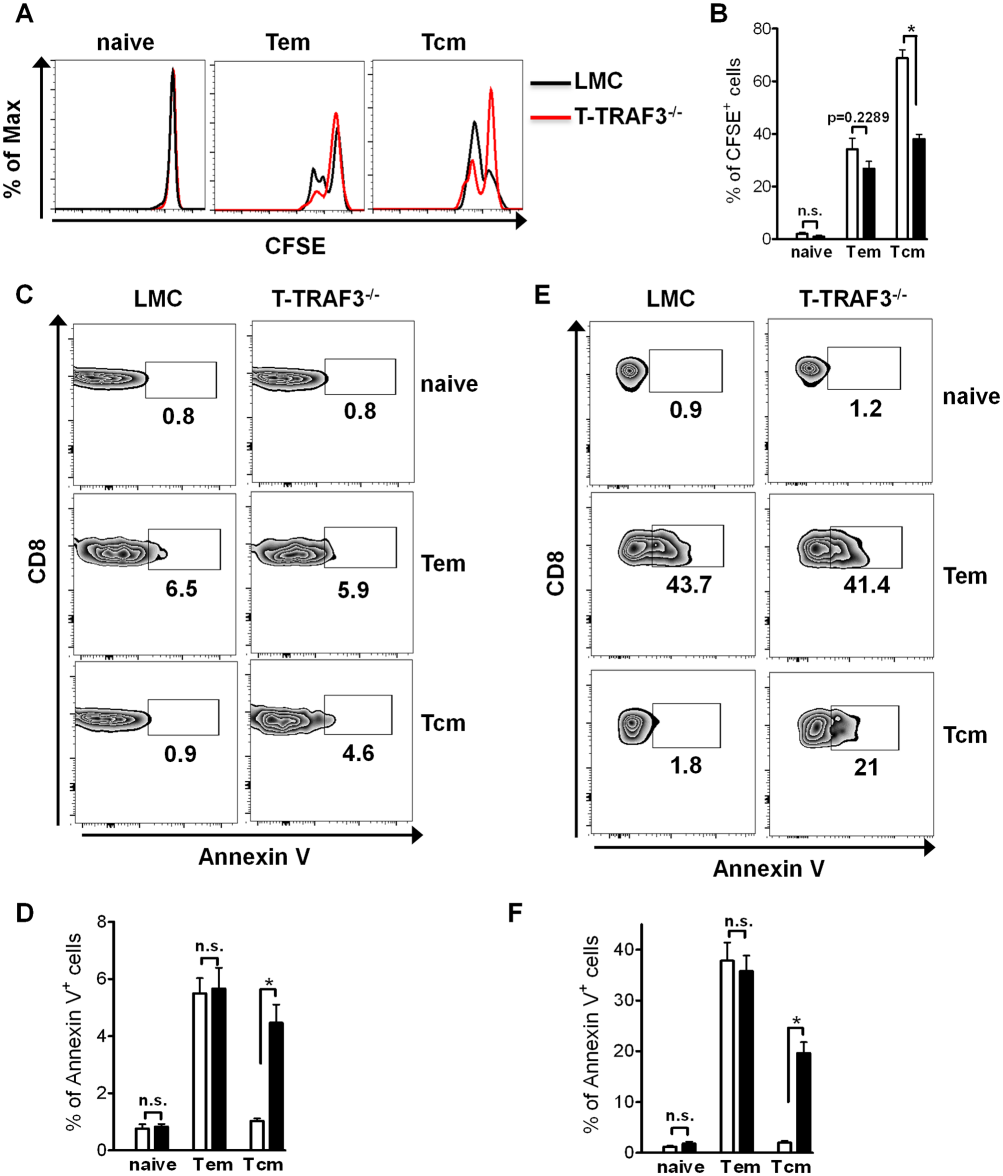
Defective homeostasis of CD8^+^ Tcm cells in the absence of TRAF3. (**A**) and (**B**) Sorted CD8^+^ T cells were labeled with CFSE and stimulated with 60 ng/ml IL-15 for 72 hr. Cells were stained for CD8, CD44 and CD62L. The dilution of CFSE in different populations is shown in (**A**), and data from three experiments are summarized in (**B**). (**C**) and (**D**) Freshly isolated splenocytes were stained for CD8, CD44, CD62L and Annexin V. Different CD8^+^ T cell subsets were gated for Annexin V analysis (**C** and **D**). Data represent 6 mice per group. (**E**) and (**F**) Splenocytes cultured for 24 hr without stimulation and stained for CD8, CD44, CD62L and Annexin V. Annexin V positive cells in different CD8^+^ T cell subsets are shown (**E** and **F**). Data represent one of three individual experiments. *p<0.01.

CD8^+^ Tcm cells are a relatively stable population, with no appreciable change in numbers with time. This suggested that altered homeostasis of this population could underlie its reduction in size in T-TRAF3^−/−^ mice. To investigate this possibility, purified CD8^+^ T cells were labelled with the fluorescent dye CFSE and cultured with 60 ng/ml IL-15 for 72 hr. Flow cytometric analysis of CFSE dilution showed that TRAF3-deficient Tcm cells exhibit significantly decreased proliferation compared to LMC Tcm cells (Figure 5A and B), consistent with impaired IL-15 signaling in the absence of TRAF3. In contrast, TRAF3-deficient Tem cells showed only slightly decreased proliferation, and no proliferation was found with naïve T cells (Figure 5A and B). To explore whether impaired IL-15 signaling also impacted Tcm cell survival, freshly isolated splenocytes were stained for Annexin V. Consistently and markedly more Tcm cells were Annexin V positive in the absence of TRAF3, indicating that TRAF3 deficient Tcm cells undergo cell death much faster than LMC Tcm cells (Figure 5C and D). Noticeably, this phenomenon was not seen in Tem and naïve T cell populations (Figure 5C and D). Consistent with this result, when purified CD8^+^ T cells were cultured *in vitro* without addition of IL-15, more cell death was also observed in TRAF3 deficient Tcm cells, but not in other populations (Figure 5E and F). Taken together, these results indicate that TRAF3 deficiency impaired CD8^+^ Tcm cell homeostasis.

## Discussion

The multiple and complex roles played by TRAF3 in T lymphocyte biology have only recently begun to be revealed [21], [23], [24]. We report here that TRAF3 plays differential roles in the responses of CD4^+^ vs CD8^+^ T cells to TCR stimulation. In addition, we found that TRAF3 is an important regulator of IL-15 mediated signaling, and thus is required for Tcm cell homeostasis and maintenance.

In T-TRAF3^−/−^ mice, TCR signaling in peripheral T cells is impaired, yet it suffices to allow normal development of conventional T cells [21], suggesting that TCR signaling is not significantly affected at the early T cell developmental stages. The finding that *in vitro* TCR-stimulated cytokine production by CD4^+^ T cell is more severely impaired than that of CD8^+^ T cells indicates that TRAF3 might play distinct roles in different T cell subsets [21]. Indeed, short-term *in vitro* TCR stimulation primarily affected activation of CD4^+^ T cells in the absence of TRAF3. However, whether and how TRAF3 affects CD8^+^ T cell responses to long-term TCR stimulation is difficult to determine, because T cell TRAF3 deficiency can adversely affect both survival in culture and cytokine production [21]. To add to the complexity, although there is markedly impaired TCR signaling in TRAF3^−/−^ CD4^+^ T cells, increased CD4^+^ effector/memory T cells were found in T-TRAF3^−/−^ mice, which initially seems contradictory. However, a very recent study found increased CD4^+^ effector/memory T cells in Foxp3^Cre^TRAF3^fl^°^x/fl^°^x^ mice, due to defective suppressive effects of TRAF3-deficienct Treg cells upon this T cell population [24]. This strongly suggests that this effect contributes to the increased CD4^+^ effector/memory T cells in T-TRAF3^−/−^ mice.

The finding that only CD8^+^ Tcm cells, but not Tem and naïve cells were reduced in T-TRAF3^−/−^ mice is of particular interest. This phenotype cannot be explained only by weakened TCR signaling in CD8^+^ T cells. Indeed, we found that IL-15 signaling in this population was also impaired. Correspondingly, the proliferation rate of Tcm cells was lower than that of LMC Tcm cells in response to IL-15 stimulation. Thus, TRAF3 is involved in IL-15 signaling and is essential for peripheral homeostatic maintenance of CD8^+^ Tcm cell subsets. This finding highlights the critical and multifacted roles for TRAF3 in the T cell biology [25]. The detailed molecular mechanisms of how TRAF3 regulates IL-15 signaling await further investigation.

In B lymphocytes, it has been demonstrated that TRAF3 interacts with TRAF2, cIAP1 and cIAP2 and negatively regulates the non-canonical NF-κB2 pathway [26], [27]. Depletion of TRAF3 results in constitutive activation of the NF-κB2 pathway in all cell types examined to date [19]–[21]. It appears that activation of the NF-κB2 pathway in stromal cells, including thymic epithelial cells and dendritic cells, plays more important roles in regulating T cell development than NF-κB2 activation in T cells per se [28]–[34]. A recent study shows that the NF-κB2 pathway is required for efficient effector/memory T cell development [35]. However, whether constitutive activation of this pathway also impacts Tcm cell development or maintenance is not clear.

In summary, our study indicates that in addition to playing important roles in T cell function, TRAF3 plays distinct roles in CD4^+^ versus CD8^+^ T cells in response to TCR stimulation, and that TRAF3 is required for normal IL-15 signaling, and hence for CD8^+^ Tcm cell homeostasis and maintenance.
